# Phytochemical profile and antimicrobial activity of essential oils from two *Syzygium* species against selected oral pathogens

**DOI:** 10.1186/s12906-023-04277-1

**Published:** 2023-12-12

**Authors:** Sahar S. Salem, Heba E. Elsayed, Samah Shabana, Mohamed T. Khazaal, Fatma A. Moharram

**Affiliations:** 1 Department of Pharmacognosy, Faculty of Pharmaceutical Sciences and Drug Manufacturing, Misr University for Science and Technology, 6th October, Giza, Egypt; 2 Department of Pharmacognosy, Faculty of Pharmacy, Helwan University. Ein Helwan, Cairo, 11795 Egypt; 3 Botany and Microbiology Department, Faculty of Science, Helwan University, Ein Helwan, Cairo, 11795 Egypt

**Keywords:** Antimicrobial, Biofilm, Essential oils, Extraction, *Syzygium*

## Abstract

**Background:**

The genus *Syzygium* (Myrtaceae) comprises several essential oil-rich species that are utilized traditionally for treating tooth infections and toothache. The current study aimed to extract essential oils (EOs) from the leaves of *Syzygium samarangense* and *Syzygium malaccense* cultivated in Egypt for the first time and screen their antimicrobial potential against oral-related pathogens.

**Methods:**

The intended EOs were extracted using hydrodistillation (HD) by boiling fresh leaves with distilled water; supercritical fluid (SF) by extracting the dried leaves using supercritical CO_2_ at 40 °C and 150 bar; and the headspace (HS) in which the fresh leaves were heated in a glass vial and the vaporized aroma were analyzed. The volatile constituents were analyzed using GC/MS and identified by comparing the experimental Kovats' retention indices with the literature. The antimicrobial activity was assessed against *Staphylococcus aureus, Enterococcus faecalis, Escherichia coli,* and *Candida albicans* using agar diffusion, microwell dilution, and biofilm formation assays. Statistical significance (*p* < 0.05) was determined by applying one-way ANOVA and Duncan's post hoc test.

**Results:**

The yield of the extracted EOs differs between the applied methods, and the SF approach harvested the maximum (0.52–0.46%). The GC–MS analysis of SF EOs revealed a discrepancy between the two species. Since *S. malaccense* showed an abundance of hydrocarbons represented mainly by squalene (60.60%), *S. samarangense* was deemed to have oxygenated sesquiterpenes exemplified in globulol (52.09%). On the other side, the HD and HS EOs were sequentially comparable, while differed in the percentage of their majors. *γ*-terpinene (33.06%) pioneered the HS-derived aroma of *S. malaccense*, while *S. samarangense* was abundant with *α*-pinene (30.18%). Concurrently, the HD EOs of *S. malaccense* and *S. samarangense* were commonly denoted by caryophyllene oxide (8.19%-18.48%), *p*-cymene (16.02%- 19.50%), and γ-terpinene (12.20%-17.84). Ultimately, both species EOs exhibited broad-spectrum antimicrobial potential, although the HD EO was more potent than the SF EO. The HD EOs of both species potently inhibited the growth of *E. coli* (MIC 3.75 µL/mL) and suppressed *C. albicans* biofilm formation by 83.43 and 87.27%, respectively. The SF-EOs efficiently suppressed the biofilm formation of Gram-positive bacteria by 76.45%-82.95%.

**Conclusion:**

EOs extracted from both species by different methods possessed a unique blend of volatile components with broad-spectrum antimicrobial activity. They were promoted as bioactive hits for controlling oral infections, however further investigations concerning their safety in clinical settings are needed.

**Supplementary Information:**

The online version contains supplementary material available at 10.1186/s12906-023-04277-1.

## Background

Essential oils (EOs) have shown great interest over the past decade as holistic integrative modalities for traditional medicinal treatments [1]. They have been used by many populations as alternatives to ready-prescribed medications because of their low toxicity, pharmacological activities, and economic viability [1]. EOs are hydrophobic, complex extracts comprised of hundreds of organic, low-molecular-weight volatile components. They could be simply categorized according to their chemical scaffold into terpenoids and aromatic, each of which is subclassified as oxygenated and non-oxygenated [2]. The variability in their chemical composition could be attributed to a set of interrelated factors such as the extraction method, geographic sources, environmental conditions, maturity stage, plant organ, and genetics [3, 4]. The unique chemical blend of volatile components enriched the EOs with a surge of therapeutic values, especially in the field of dentistry and oral hygiene. For instance, several studies have documented the beneficial effect of supplementing mouthwashes with EOs in inhibiting plaque formation, diminishing anxiety, and reducing toothache [5]. Also, dressings treated with EOs enhanced the healing of wounds after oral surgery. Meanwhile, treating the surface of dental implants with EOs prevented biofilm formation and showed more potent inhibitory activity against microbiomes than methylparaben [5]. The antimicrobial potential is urgently essential because, recently, many synthetic antimicrobial drugs have been facing treatment limitations due to the development of antimicrobial drug resistance and acute toxicity [6]. In this regard, the exploitation of new EOs to control multidrug-resistant pathogenic microorganisms can help combat various infectious diseases. Several plant species are well-known for their notable EO content, among them are those of the genus Syzygium [7, 8]. *Syzygium* is a genus in the myrtle family (Myrtaceae) that comprises about 1193 recognized aromatic species worldwide [7]. *Syzygium* is restricted to tropical and subtropical regions of the Old World [7]. The genus has unprecedented culinary use, as witnessed by the unexpanded flower buds of *S*. *aromaticum* (Clove), which are the most significant economic spices [8]. Traditionally, clove essential oil is used as a pain reliever in dental care as well as in treating tooth infections [8]. Other Syzygium species produce edible fruits that are eaten fresh or used commercially such as, *S. malaccense* and *S.* samarangense [7]. *Syzygium malaccense* (L.) Merr. & L. M. Perry, known as the Malaysian apple or Malay apple, is an evergreen, flowering tree with edible fruits native to the forested lowlands of Malaysia, Southeast Asia, and Australia [9]. Traditionally, the leaves are chewed, or the juice is dripped into the mouth of infants to treat mouth infections and oral thrush [10]. In Malaysia, the dried leaves are used on a cracked tongue, while bark extract is used for abdominal ailments, coughs, and sore throats [10]. In the same context, *Syzygium samarangense* (Blume) Merr. & L. M. Perry, recognized as wax apple or java apple, is a tropical non-climactic tree found in south to southeast Asia, Taiwan, and other tropical countries [11]. The species is a well-known antioxidant, immunomodulatory, antibacterial, anticancer, anti-inflammatory, analgesic, and antidepressant [11]. Both species promise to support nutritional values and human health, yet the antimicrobial rationale of their EOs in oral infections has not been studied.

The oral cavity harbors diverse microbiomes that, under normal conditions, reside in homeostasis [12]. The imbalance of these microbiomes or their colonization with new microorganisms of viral, fungal, or bacterial origin can infect the oral cavity and its mucosa, influencing oral health and causing several diseases [13]. Dental biofilms are formed due to the attachment of oral microbiomes to the hard and soft tissues of the oral cavity. They are highly associated, embedded in an extracellular matrix [14], and responsible for many oral diseases. Meanwhile, the incidence of primary bacterial infections of the oral mucosa is rare because of the protective role of the epithelium layer, the saliva's antibacterial characteristics, and the immune responses of the phagocytes [13]. However, if the oral mucosa is disrupted due to poor oral hygiene, trauma, smoking, alcohol misuse, or any other stimuli, the risk of primary bacterial infections will be hazardous. Among common oral microbiomes are *Enterococcus faecalis, Staphylococcus aureus, Escherichia coli*, and *Candida albicans*. *E. faecalis* is an obligate anaerobe that has been related to caries, endodontic infections, and peri-implantitis [15, 16]. Even though *Enterococci* are sensitive to some antibiotics the emergence of multidrug-resistant strains is becoming a matter of concern. *S. aureus* is another favorable inhabitant of the oral cavity and has been added to the drug-resistant microbiomes due to the excessive prophylactic usage of antibiotics [17, 18]. On the other side, *E. coli* has been frequently reported from acute dental abscesses, ranging from 0.7% to 15% [19]. Lastly, *C. albicans* is a ubiquitous commensal organism and by far the principal causative agent of oral candidiasis accounting for up to 95% of cases [20].

Given the incidence of oral infections, increased resistance by microbiomes to antibiotics, adverse effects of some antibacterial agents, and financial considerations in developing countries, there is a need for alternative options. Constituting mainly effective, safe, and economical advantages. These unique features were collectively documented for natural phytochemicals, just like essential oils.

In the current study, the GC profile of the EOs extracted by three different methods from *S.* *samarangense* and *S. malaccense* leaves cultivated in Egypt were comparatively investigated for the first time. Moreover, the ability of the extracted EOs to inhibit the growth of oral-related pathogens and reduce their ability to develop oral biofilm was also examined.

## Material and methods

### Plant material

The leaves of *Syzygium malaccense* (L.) Merr. & L. M. Perry and *Syzygium samarangense* (Blume) Merr. & L. M. Perry were collected before the flowering stage, from June to August 2021, at Mazhar Botanical Garden. The plants were collected after the garden authorities’ permission following the local garden`s guidelines for collection and complying with the collection legislation of Egypt. The plants’ names were verified by Dr. Trease Labib, Senior Botanist at Mazhar Botanical Garden, Cairo, Egypt. Voucher specimens of each species (01Sma, 2021, and 01Ssa, 2021) were collected and deposited at the herbarium of the Pharmacognosy Department, Faculty of Pharmacy, Helwan University, Cairo, Egypt.

### Extraction of the essential oils 

#### Hydrodistillation (HD)

Conventional hydrodistillation was performed following the protocol reported previously [21]. In short, 300 g fresh leaves of each species were cut into small pieces, immersed with distilled water in a 2 L extraction flask of Clevenger apparatus, and brought to a boil for at least 5 h. The oil layer was collected and filtered over anhydrous sodium sulfate to remove any residual moisture. The extraction protocol was repeated twice to obtain enough volume of each oil.

#### Supercritical fluid (SF)

Supercritical CO_2_ was used for the extraction of essential oils following the protocol and conditions described by Rodriguez and co-workers [22] and Elsayed et al. [21]. Briefly, about 400 g of air-dried leaves of both species were extracted separately at 40 °C and 150 bar using Speed TM SFE-2/4, Applied separations, built in conjunction with the USDA1 (United States) laboratory scale apparatus charged with supercritical carbon dioxide (SCC). The flow rate of the SCC was adjusted as static at a rate of 10 mL/min for 1 h, while in dynamic mode for 1 h with a total processing time of 3 h. Absolute ethanol, as a modifier, was added with a flow rate of 0.2 mL/min. The extraction protocol was repeated twice to obtain enough amount of each oil. Ultimately, the collected oils in HD and SFE were stored in amber, sealed vials at 4 °C until GC/MS analysis. The percentage of the extracted oil was calculated as follows: oil volume (mL) /100 g of plant material.

#### Headspace (HS)

The headspace micro-extraction of the leaves’ volatile constituents was achieved following the protocol described by Ibrahim et al. [23]. About 2 g of leaves, from each plant species, were added to a 5 mL glass vial at 60–70 °C. The vaporized constituents were directly analyzed using a headspace sampler HS-20 coupled to a Shimadzu GCMS-QP2020 gas chromatograph mass spectrometer (Koyoto, Japan) following the conditions stated previously [23].

### Gas chromatography/ Mass spectrometry (GC–MS) analysis

The GC/MS analysis volatile constituents were measured on Shimadzu GCMS-QP2010 (Koyoto, Japan) linked to a quadrupole mass spectrometer (Shimadzu Corporation, Kyoto, Japan). Rtx-5MS fused bonded column (30 m × 0.25 mm internal diameter × 0.25 µm film thickness) (Restek, United States) was adopted for the chromatographic separation of each sample. Diluted samples (1% v/v) were injected with split mode (split ratio 1: 15). The separation operated primarily at 45 °C for 2 min (isothermal), then increased gradually to 300 °C at a rate of 5 °C/min. At the same time, the injector temperature was kept at 250 °C. The Helium carrier gas flow rate was 1.41 mL/min. All the mass spectra were recorded by a flame ionization detector following the standard conditions for the filament emission current, 60 mA; ionization voltage, 70 eV; ion source, 200 °C.

### Identification of volatile oil components

The acquired volatile constituents were recognized by matching their Kovats' retention indices (RI) with that of *the n*-alkanes standard series (C_8_-C_28_). In addition, their mass spectra were compared with those reported in the NIST (National Institute of Standards and Technology) and Wiley mass spectral database (similarity index > 90%).

### In vitro antimicrobial activity

#### Standard microbes, antibiotics, and culture media

The stock cultures of *Staphylococcus aureus* ATCC 25923*, Enterococcus faecalis* ATCC 29212*, Escherichia coli* ATCC 8739*,* and *Candida albicans* ATCC 10231 were obtained from Microlab, Institute of Research and Technology, Vellore, Tamilnadu, India. The choice of these reference strains depends on their association with oral infections and diseases, making them suitable for evaluating the antimicrobial effectiveness of the extracted EOs in combating oral pathogens. Mueller–Hinton agar (MHA) and broth (MHB), biological grade sterile DMSO, and standard antibiotic discs (6.0 mm) were purchased from Oxoid, Thermo Fisher Scientific (MA, United States).

#### Susceptibility test

It was carried out using the agar well-diffusion method according to the Clinical and Laboratory Standards Institute [24–26], Gholizadeh et al. [27], and Balouiri et al. [28]. In short, 100 µL of each reference strain (1 × 10^5^ CFU/mL) was spread separately over the MHA medium. After the media solidified, wells were made by 0.6 cm sterile cork-borer to receive 50 µL of different concentrations of the extracted HD EOs (0–50 µL/mL) or SFC EOs (0–13.7 mg/mL). The plates were first kept in the refrigerator for 30 min to facilitate the diffusion of the sample extracts into the agar [29, 30], followed by incubation at 37 °C/24 h and 25 °C/ 5 d for bacteria and fungus strains [31], respectively. The antimicrobial susceptibility of each sample was determined by measuring the developed zones of inhibition (ZOI) diameter in mm. The activity was compared to conventional antibiotics with different modes of action, including amikacin (AK, 30 μg/mL), amoxicillin (AX, 25 μg/mL), ampicillin/sulbactam (SAM, 20 μg/mL), norfloxacin (NOR, 10 μg/mL), ofloxacin (OFX, 5 μg/mL), and nystatin (NS, 50 µg/ml). Sterile DMSO was used as a negative control.

#### Determination of minimum inhibitory concentrations (MICs)

The broth microdilution assay was adopted to determine the MICs of each tested sample [24, 27, 32]. Stock solutions of *S. malaccense* HD, *S. samarangense* HD, *S. malaccense* SF, and *S. samarangense* SF EO samples were prepared by dissolving 50 µL, 30 µL, 11.7 mg, and 13.7 mg, respectively, in 1 mL DMSO. Each EO stock solution was diluted to 1/10 in sterile MHB media. Thereafter, 100 µL of MHB was added in each well (2 to 12), while 150 µL of each 1/10 diluted sample was added in the first column of microtiter plates. Subsequently, two-fold dilution was done by transferring 100 µL from the first to the 11^th^ well. Lastly, 100 µL of each microbial culture (1 × 10^5^ CFU/mL) was added to each well except the last one which is considered blank. All microtiter plates were incubated at 37 °C/24 h and 25 °C/ 5 d for inoculated bacteria and fungus strains, respectively. Absorbances were measured at λ_max_ 620 nm using an automated microplate reader (ChroMate 4300, United States) and represented graphically using Microsoft Excel version 2019. Statistical significance was determined using a *p*-value of < 0.05 compared to the control group.

#### Antibiofilm formation quantitative assay

The antibiofilm formation was explored following the protocol stated in the literature [33, 34] with some modifications. Briefly, different concentrations of the extracted HD EOs (0–50 µL/mL) or SFC EOs (0–13.7 mg/mL) were added to a 96-well polystyrene plate followed by the addition of 100 μL microbial suspension (1 × 10^5^ CFU/mL). The plates were incubated statically at 37 °C for 48 h then washed twice with sterile PBS (1X, pH 7.3), and air dried. 400 μl methanol was added for 15 min for fixation of biofilm. The fixed biofilms were stained with 1% crystal violet solution for 10 min. Finally, absolute ethanol was added to each well, and the absorbance was read spectrophotometrically at 630 nm using a microplate reader (ChroMate 4300, United States).

### Statistical analysis

The optical density reduction through viability % and biofilm % associated with each tested sample was compared with that of untreated control by one way ANOVA using Excel version 2019 and Duncan’s post hoc test using IBM SPSS version 20 software.

## Results

### Extraction and characterization of volatile constituents

Volatile constituents from the leaves of *S.* *samarangense* and *S. malaccense*, cultivated in Egypt, were extracted using three methods *viz*, hydrodistillation (HD), supercritical fluid (SF), and headspace (HS). The HD afforded a pale-yellow EOs, with a faint fruity odor constituting 0.20% and 0.16% for *S.* *samarangense* and *S. malaccense*, respectively while SF showed up a brown, viscous, EOs with a 0.52% and 0.46% for *S.* *samarangense* and *S. malaccense*, respectively. As observed, the implemented extraction methods displayed significant differences in their yield, while the yield from the same extraction method was quite comparable among different species. Meanwhile, the volatile constituents derived from the HS method were unrecoverable, which is one of its common drawbacks. All derived volatile constituents were analyzed qualitatively and quantitatively using hyphenated GC/MS and their abundances were estimated by GC/FID (Supplementary Figures S1-S6). The results of the GC/MS analysis of *S. malaccense* volatile constituents (Table 1) showed the names of the compounds, which constituted 85.48%, 98.93%, and 66.39% of the total identified components in the HD, HS, and SF-derived oils and aroma, respectively. As a general observation, volatile constituents from HS and SF extraction methods are, by far, pioneered by non-oxygenated compounds calculated as 96.45% and 65.93%, respectively. Specifically, monoterpene hydrocarbons (MH) amounted to 96.44% of the HS-aroma with *γ*-terpinene (33.06%) and *β*-pinene (19.75%) are the major volatiles, while the triterpenoid hydrocarbon, squalene made up nearly 66.60% of the SF-derived oil. In a dissimilar way, the HD-EO was abundant with oxygenated derivatives (47.39%) in comparison to non-oxygenated ones (38.09%). The oxygenated sesquiterpenes (OS) constituted a higher percentage (43.10%) in comparison to oxygenated monoterpenes (4.08%) with caryophyllene oxide (18.48%) and *tau*-cardinal (11.99%) being the major OS, while *p*-cymene (16.02%) and *γ*-terpinene (12. 2%) were the most abundant MH.

**Table 1:** Identified volatile components in *Syzygium malaccense* leaves obtained by hydrodistillation (HD), head space (HS), and supercritical fluid (SF) methods

**No**	**R**_**t**_	**Compound name**	**MF**	**RI**_**exp**_	**RI**_**lit**_	**Concentration %**		
						**HD**	**HS**	**SF**
1	4.92	2-Hexenal	C_6_H_10_O	828	832	nd	1.14	nd
2	6.51	Butyl glycol	C_6_H_14_O_2_	886	903	0.21	nd	nd
3	7.50	*α*-Thujene	C_10_H_16_	922	921	1.30	13.79	nd
4	7.67	*α*-Pinene	C_10_H_16_	928	929	4.91	10.38	nd
5	8.02	Camphene	C_10_H_16_	941	941	nd	0.02	nd
6	8.84	*β*-Pinene	C_10_H_16_	971	973	nd	19.75	nd
7	9.24	Octanal	C_8_H_16_O	985	1000	nd	0.02	nd
8	9.66	*α*-Phellandrene	C_10_H_16_	1000	1002	nd	3.20	nd
9	10.05	*α*-Terpinene	C_10_H_16_	1013	1015	2.24	8.24	nd
10	10.14	*p*-Cymene	C_10_H_14_	1016	1016	16.02	7.07	nd
11	10.43	Limonene	C_10_H_16_	1025	1025	0.18	0.93	nd
12	11.37	*γ*-Terpinene	C_10_H_16_	1055	1059	12.20	33.06	nd
13	14.73	Terpinen-4-ol	C_10_H_18_O	1164	1168	3.90	0.75	nd
14	15.06	*α*-Terpineol	C_10_H_18_O	1174	1179	nd	0.15	nd
15	17.27	Methyl citronellate	C_11_H_20_O_2_	1249	1250	nd	0.03	nd
16	18.21	Thymol	C_10_H_14_O	1282	1288	0.18	nd	nd
17	22.00	Caryophyllene	C_15_H_24_	1416	1416	1.24	0.10	2.74
18	22.85	Humulene	C_15_H_24_	1440	1445	nd	nd	0.57
19	24.56	Cubenene	C_15_H_24_	1505	1522	nd	nd	2.02
20	25.79	Caryophyllene oxide	C_15_H_24_O	1564	1565	18.48	nd	nd
21	25.90	Globulol	C_15_H_26_O	1568	1568	2.64	nd	nd
22	26.07	Viridiflorol	C_15_H_26_O	1575	1580	0.47	nd	nd
23	27.17	*tau*-Cadinol	C_15_H_26_O	1620	1625	11.99	nd	nd
24	27.52	*α*-Cadinol	C_15_H_26_O	1632	1632	9.52	nd	0.46
25	49.03	Squalene	C_30_H_50_	2796	2814	nd	nd	60.6
Total identified compounds						85.48	98.93	66.39
Non-oxygenated compounds						38.09	96.45	65.93
Monoterpenes						36.85	96.44	-
Sesquiterpenes						1.24	0.10	5.33
Triterpenes						-	-	60.60
Oxygenated compounds						47.39	2.09	0.46
Monoterpenes						4.08	0.90	-
Sesquiterpenes						43.10	-	0.46
Others						0.21	1.16	-

*R*_*t*_ Retention time*, MF* Molecular formula, *RI*_*Exp*_ Experimental retention index, *RI*_*Lit*_ Reference retention index, *nd* Not detectable

On the other side, Table 2 displayed the identified volatile components in *S. samarangense* which constituted about 81.73%, 95.82%, and 69.72% of the total identified volatiles in HD, HS, and SF, respectively. Interestingly, SF-EO was abundant in oxygenated volatiles (57.91%), while HD-EO and HS were rich in non-oxygenated compounds (55.45% and 83.19%, respectively). There is a discrepancy in the most abundant component in each EO, for example, globulol (52.09%) represents the major OS in the case of SF-EO while HD-EO and HS are rich by MH being 51.91% and 80.48%, respectively. *p*-Cymene (19.50) and γ-terpinene (19.50%) represented the major MH in HD oil while *α-*pinene and γ-terpinene (22. 4%) are the major MH for HS. Furthermore, caryophyllene oxide (8.19%) and *tau*-cadinol (10.56%) represented the major OS in the case of HD oil. To sum up our key findings, there is a significant difference in the chemical composition of the EOs of the two species extracted by the SF method compared to the volatile components obtained by the HD and HS. The SF-EO of *S. samarangense* could be deemed a source for the oxygenated sesquiterpene globulol, as it constitutes beyond 50% of its total identified components. Meanwhile, the SF-EO of *S. malaccense* could be a resource for the hydrocarbon squalene (> 60%). On the other side, HD and HS-derived EOs of both species showed significant differences mainly in the percentage of their major identified volatile components, as exemplified in the calculated percentage of caryophyllene oxide, *γ*-terpinene, and *α*-pinene (Tables 1 and 2). That in part may suggest the preference of one species rather than the other if seeking for specific component concentration.

**Table 2:** Identified volatile components in *Syzygium samarangense* leaves obtained by hydrodistillation (HD), head space (HS), and supercritical fluid (SF) methods

**No**	**R**_t_	**Compound name**	**MF**	**RI**_exp_	**RI**_lit_	**Concentration %**		
						**HD**	**HS**	**SF**
**1**	4.93	2-Hexenal	C_6_H_10_O	828	832	nd	10.22	nd
**2**	5.53	2-Hexenol	C_6_H_12_O	850	854	nd	0.90	nd
**3**	5.63	1-Hexanol	C_6_H_14_O	854	861	nd	0.85	nd
**4**	7.49	*α* -Thujene	C_10_H_16_	922	926	2.15	16.47	nd
**5**	7.67	*α-*Pinene	C_10_H_16_	928	929	7.89	30.18	nd
**6**	9.31	*β*-Pinene	C_10_H_16_	988	985	0.32	0.93	nd
**7**	9.66	*α*-Phellandrene	C_10_H_16_	1000	1000	0.50	1.77	nd
**8**	10.05	*α* -Terpinene	C_10_H_16_	1013	1015	3.33	3.86	nd
**9**	10.13	*p*-Cymene	C_10_H_14_	1016	1016	19.50	4.87	nd
**10**	10.41	Sylvestrene	C_10_H_16_	1025	1026	0.32	nd	nd
**11**	10.44	1,8-Cineole	C_10_H_18_O	1023	1027	nd	0.66	nd
**12**	11.02	3-Carene	C_10_H_16_	1044	1031	0.06	nd	nd
**13**	11.31	*γ*-Terpinene	C_10_H_16_	1054	1059	17.84	22.40	nd
**14**	14.71	Terpinen-4-ol	C_10_H_18_O	1163	1168	3.16	nd	nd
**15**	15.04	*α* -Terpineol	C_10_H_18_O	1173	1173	0.47	nd	nd
**16**	17.24	Methylcitronellate	C_11_H_20_O_2_	1248	1250	0.07	nd	nd
**17**	17.79	Carvacrol	C_10_H_14_O	1267	1279	0.24	nd	nd
**18**	20.87	Ylangene	C_15_H_24_	1375	1375	0.08	nd	nd
**19**	21.24	*α*-Guaiene	C_15_H_24_	1380	1409	0.32	nd	7.06
**20**	22.01	Caryophyllene	C_15_H_24_	1417	1418	2.28	2.71	4.75
**21**	22.81	Humulene	C_15_H_24_	1448	1450	0.21	nd	nd
**22**	23.37	*γ*-Muurolene	C_15_H_24_	1470	1470	0.52	nd	nd
**23**	23.96	*α*-Muurolene	C_15_H_24_	1493	1494	0.13	nd	nd
**24**	25.53	Caryophyllenyl alcohol	C_15_H_26_O	1554	1557	0.16	nd	nd
**25**	25.84	Caryophyllene oxide	C_15_H_24_O	1557	1563	8.19	nd	4.04
**26**	25.90	Globulol	C_15_H_26_O	1569	1569	3.09	nd	52.09
**27**	26.08	Viridiflorol	C_15_H_26_O	1575	1580	0.34	nd	nd
**28**	27.18	*tau-*Cadinol	C_15_H_26_O	1621	1625	10.56	nd	nd
**29**	28.99	*β*-santalol	C_15_H_24_O	1689	1689	-	nd	1.78
Total identified compounds						81.73	95.82	69.72
Non-oxygenated compounds						55.45	83.19	11.81
Monoterpenes						51.91	80.48	-
Sesquiterpenes						3.54	2.71	11.81
Oxygenated compounds						26.28	12.63	57.91
Monoterpenes						3.94	0.66	-
Sesquiterpenes						22.34	-	57.91
Others						-	11.97	-

*Rt *Retention time*, MF* Molecular formula, *RI*_*Exp*_ Experimental retention index, *RI*_*Lit*_ Reference retention index, *nd* Not detectable

### In vitro antimicrobial activity

In the current study, the antimicrobial activity of the HD and SF-EOs was investigated against four common oral pathogens namely; *S.aureus*, *E. faecalis*, *E. coli*, and *C. albicans.* The results were inferred from measuring the developed zones of inhibition (ZOI, in mm) in the agar well-diffusion assay, estimating the minimum inhibitory concentrations (MICs) in the broth microdilution assay, and ultimately inhibiting biofilm formation using crystal violet assay.

#### Agar diffusion assay

Results delineated in Table 3 explicitly show the susceptibility of Gram’s positive bacteria, *S. aureus* ATCC 25923 and *E. faecalis* ATCC 29212, to the HD-EOs especially that of *S. samarangense* with a measured zone of inhibition (ZOI) of 15–18 mm at the maximum tested dose (30 μL/mL) compared to 22 mm by the standard antibiotic Amikacin (30 μg/mL). *C. albicans* was highly susceptible to the HD-EO of *S.* *samarangense* only at 30 μL/mL with developed ZOI equivalent to 16 mm while being resistant to the standard Nystatin. Meanwhile, *S. samarangense* SF-EO inhibited the growth of all tested microbial strains (ZOI = 8–10 mm) only at the highest tested concentration (13.7 mg/mL). The effect was less promising with the *S. malaccense* as its HD-EO displayed growth inhibition to *S. aureus* and *E. faecalis* with ZOI equivalent to 8–17 mm and 16 mm, respectively, while its SF-EO inhibited the growth of *E. faecalis* with 11 mm ZOI at 11.7 mg/mL. The strength of the inhibitory effect was deduced as compared to a panel of standard antibiotic discs that were used as positive controls in the same experiment.

**Table 3:** Zones of inhibition of the tested essential oils extracted from *Syzygium samarangense* and *Syzygium malaccense* leaves by hydrodistillation (HD) and supercritical fluid (SF) methods

**Sample**	***S. samarangense*** **HD**			***S. malaccense*** **HD**				***S. samarangense*** **SF**				***S. malaccense*** **SF**				**AK**	**AX**	**NOR**	**OFX**	**SAM**	**NS**
	**µL/mL**							**µg/mL**								**µg/mL**					
**Conc.**	**30**	**20**	**10**	**50**	**30**	**20**	**10**	**13.7**^a^	**30**	**20**	**10**	**11.7**^a^	**30**	**20**	**10**	**30**	**25**	**10**	**5**	**20**	**50**
***S. aureus***	18	14	7	17	8	-	-	8	-	-	-	-	-	-	-	22	21	25	27	24	NT
***E. faecalis***	15	10	7	16	16	-	-	8	-	-	-	11	9	7		22	21	25	27	24	NT
***E. coli***	-	-	-	-	-	-	-	9	-	-	-	-	-	-	-	22	-	30	29	19	NT
***C.albicans***	16	-	-	-	-	-	-	10	-	-	-	-	-	-	-	NT	NT	NT	NT	NT	-

- Not detected, *NT* not tested, ^a^conc. in mg/mL, ZOI measured in mm, (AK); Amikacin, (AX); Amoxicillin, (NOR); Norfloxacin, (OFX); Ofloxacin, (SAM); sulbactam/Ampicillin, and (NSl); Nystatin. Results were expressed as Mean (*n* = 3) with SD ± 1

#### Well microdilution assay

**Fig. 1:**
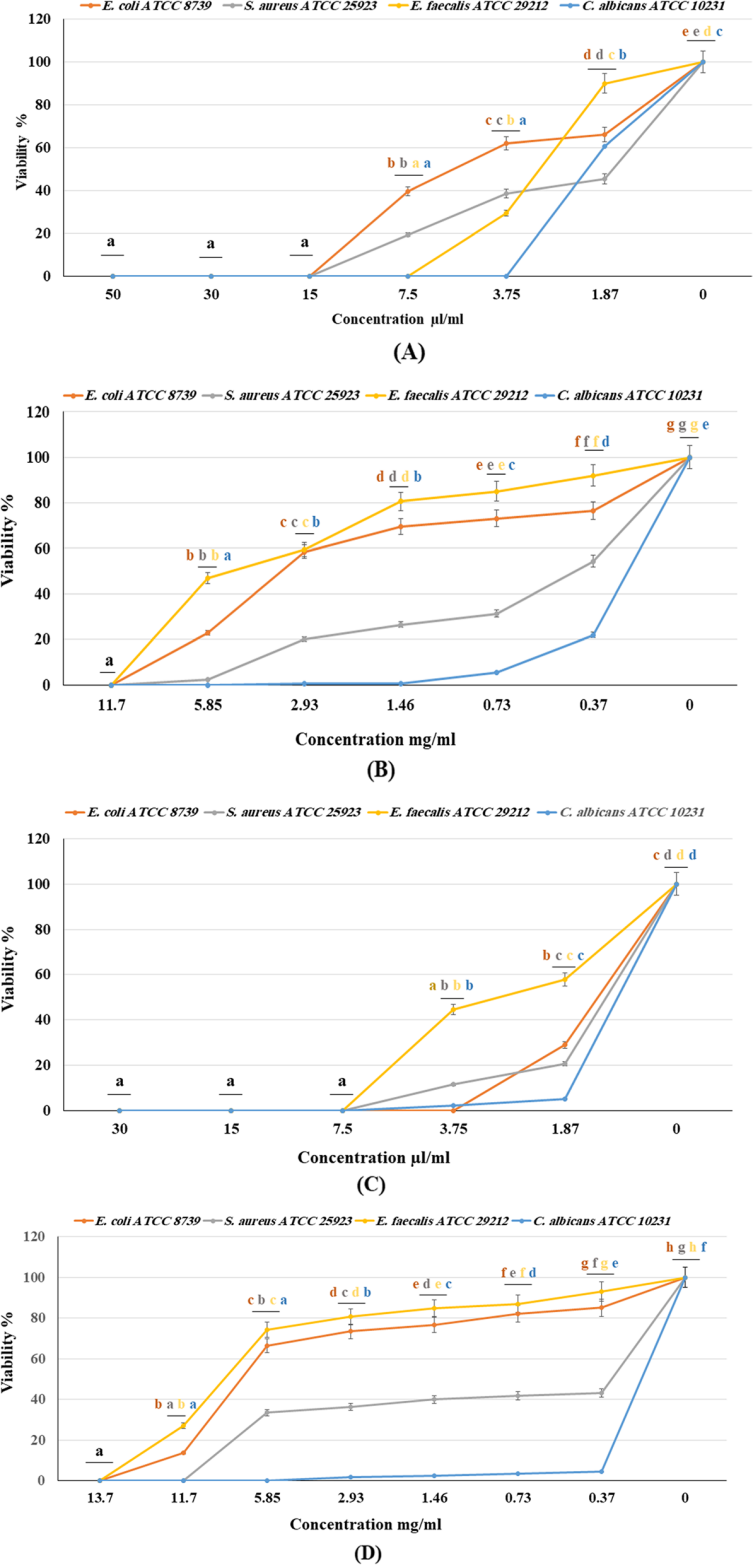
Dose–response effect of (**A**) *Syzygium malaccense* hydrodistilled essential oil; (**B**) *Syzygium malaccense* supercritical fluid extract; (**C**) *Syzygium samarangense* hydrodistilled essential oil; (**D**) *Syzygium samarangense* supercritical fluid extract on viability (%) of selected oral reference strains in broth dilution assay. Error bars represent standard deviations from the means (*n* = 3). Means with different letters are significantly different (*p* < 0.0001) by Duncan’s post hoc test

The MIC results (Table 4, Fig. 1) showed the promising ability of the tested EOs to suppress the growth of the selected oral microbial strains with variable potency. Interestingly, the HD-EO from *S. samarangense* and *S. malaccense* were the most effective and exhibited potent growth inhibition to *S. aureus* ATCC 25923 (MICs = 7.5 & 15 µL/mL), *E. faecalis* ATCC 29212 (MICs = 7.5 & 7.5 µL/mL), *E. coli* ATCC 8739 (MICs = 3.75 & 15 µL/mL), and *C. albicans* ATCC 10231 (MICs = 7.5 & 3.75 µL/mL), respectively. Meanwhile, the SF-EO, from *S. malaccense,* was more effective than *S. samarangense* against *E. faecalis* ATCC 29212 and *E. coli* ATCC 8739*,* with a MIC of 11.7 mg/mL. Additionally, the SF-EOs, from both species’, displayed equivalent MICs (11.7 and 5.85 mg/mL) against *S. aureus* ATCC 25923 and *C. albicans* ATCC 10231, respectively, compared to 100 µg/mL for the standard drug, amoxicillin.

**Table 4:** Minimum inhibitory concentrations (MICs) of the tested essential oils extracted from *Syzygium samarangense* and *Syzygium malaccense* leaves by hydrodistillation (HD) and supercritical fluid (SF) methods

	**Conc. µL/mL**		**Conc. mg/mL**		**Reference Drug**
**Reference strains**	***S. samarangense* HD**	***S. malaccense* HD**	***S. samarangense* SF**	***S. malaccense* SF**	**Amoxicillin (100 µg/mL)**
***S. aureus***	7.5	15	11.7	11.7	50
***E. faecalis***	7.5	7.5	13.7	11.7	50
***E. coli***	3.75	15	13.7	11.7	˃100
***C. albicans***	7.5	3.75	5.85	5.85	˃100

#### Biofilm formation assay

**Fig. 2:**
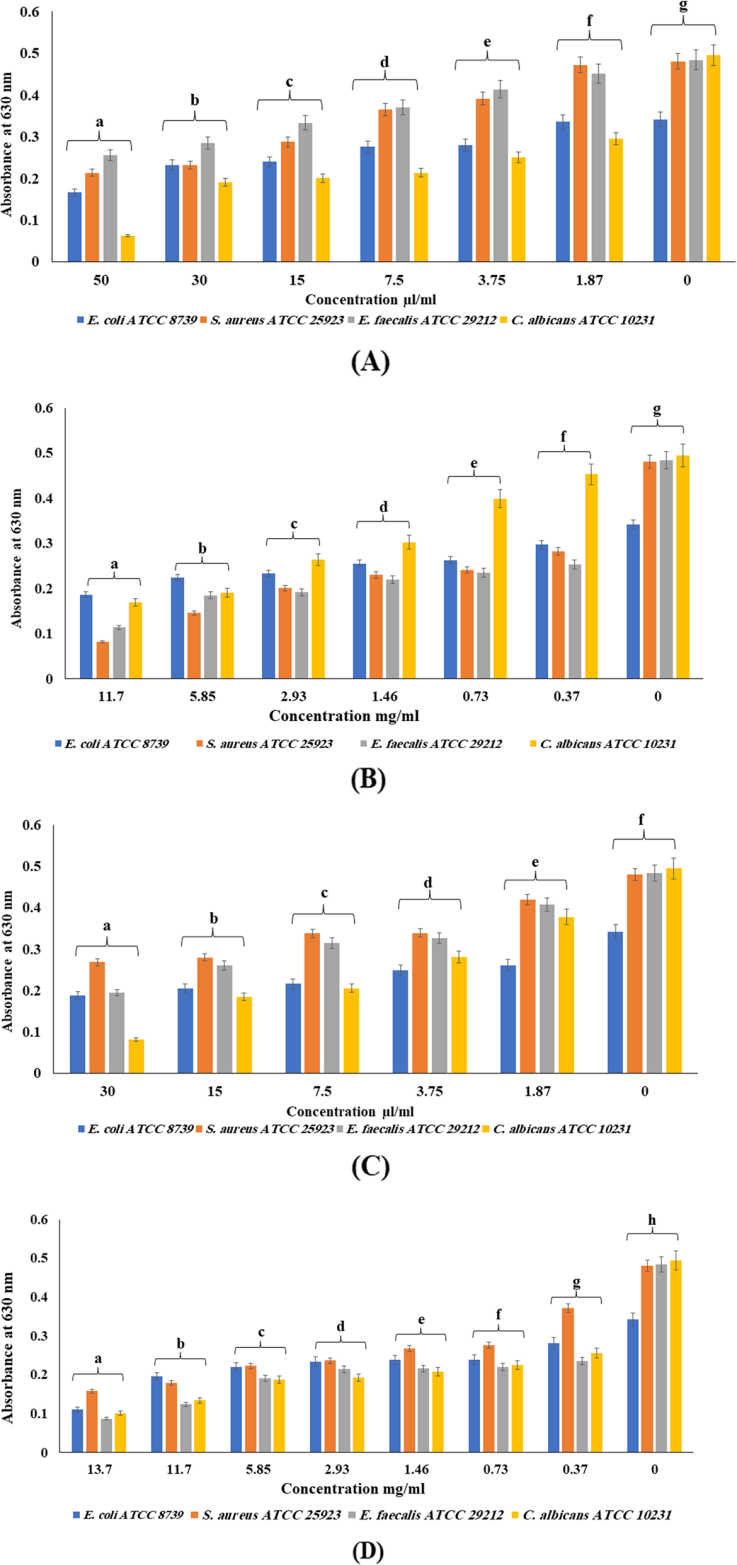
Dose–response effect of (**A**) *Syzygium malaccense* hydrodistilled essential oil; (**B**) *Syzygium malaccense* supercritical fluid essential oil; (**C**) *Syzygium samarangense* hydrodistilled essential oil; (**D**) *Syzygium samarangense* supercritical fluid essential oil on selected oral reference strains in biofilm formation assay. Error bars represent standard deviations from the means (*n* = 3). Means with different letters are significantly different (*p* < 0.0001) by Duncan’s post hoc test

The anti-biofilm formation results (Tables 5 and 6, Fig. 2) showed that the percentage of inhibiting biofilm formation by microbial strains was significantly (*p* < 0.001) reinforced by increasing the concentration of the tested EOs. It is worth noticing that HD-EOs are more potent to *C. albicans* (ATCC 10231), while SF-EOs favorably inhibit the biofilm formation by Gram-positive bacteria rather than Gram-negative strains. Briefly, the highest biofilm inhibition percentage of 87.27% was observed for *C. albicans* by the S*. malaccense* HD-EO at 50 µL/mL, followed by 83.43% at 30 µL/mL of *S. samarangense* HD. However, *S. malaccense* and *S. samarangense* SF-EOs recorded comparable potent biofilm inhibitory activity at 82.95% and 82.02% on *S. aureus,* and *E. faecalis* at the maximum tested doses, respectively. For all tested EOs, an average of 48% biofilm inhibition was exerted against *E. coli* and the maximum potency was 67.54%.

**Table 5:** Percentage of biofilm formation inhibition of the extracted essential oils from *Syzygium samarangense* and *Syzygium malaccense* leaves by hydrodistillation (HD) (*p* < 0.001)

**Tested samples**	**Conc. µL/mL**										
	***S. samarangense* HD**					***S. malaccense*** **HD**					
**Reference strains**	**30**	**15**	**7.5**	**3.75**	**1.87**	**50**	**30**	**15**	**7.5**	**3.75**	**1.87**
***S. aureus***	44.07	41.79	29.73	29.52	12.68	55.51	51.77	40.12	23.91	18.50	1.87
***E. faecalis***	59.71	46.07	34.92	32.44	15.70	46.90	41.12	30.99	23.35	14.46	6.82
***E. coli***	45.03	39.77	36.26	26.90	23.39	51.17	31.87	29.82	19.30	18.13	1.75
***C. albicans***	83.43	62.42	58.38	43.23	23.64	87.27	61.41	59.39	56.77	49.29	40.40

**Table 6:** Percentage of biofilm formation inhibition of the extracted essential oils from *Syzygium samarangense* and *Syzygium malaccense* leaves by supercritical fluid (SF) (*p* < 0.001)

**Tested samples**	**Conc. mg/mL**												
	***S. samarangense* SF**							***S. malaccense* SF**					
**Reference strains**	**13.7**	**11.7**	**5.85**	**2.93**	**1.46**	**0.73**	**0.37**	**11.7**	**5.85**	**2.93**	**1.46**	**0.73**	**0.37**
***S. aureus***	67.15	62.79	53.64	50.94	44.28	42.62	22.66	82.95	69.65	58.21	52.18	49.90	41.37
***E. faecalis***	82.02	74.38	60.54	55.79	55.37	54.55	51.45	76.45	61.78	60.33	54.55	51.45	47.73
***E. coli***	67.54	42.69	35.67	31.58	30.41	30.12	17.84	45.32	34.21	31.58	25.44	23.10	13.16
***C. albicans***	79.60	72.93	62.02	61.01	57.98	54.55	48.28	65.66	61.41	46.67	38.79	19.39	8.48

## Discussion

The oral cavity is a common inhabitant of diverse microbiomes that play a fundamental role in overall human health. Imbalances in the microbiome environment shift them to a pathogenic state causing various oral diseases such as dental cavities, gingivitis, candidiasis, and even systemic infection [35]. Despite the availability of various synthetic antimicrobial agents, the emergence of antibiotic-resistant bacteria is an emerging challenge [36]. On the other side, essential oils (EOs) have been studied for many years as potential antimicrobial agents, and several populations still applying them as traditional medicine. More than 3000 EOs have been established to be consumed, and their benefits are being progressively studied due to the need for alternative therapies for resistant oral microbiomes [37]. In the current study, two EO-rich Syzygium species, *viz*, *S.* *samarangense* and *S. malaccense*, have been investigated for their antimicrobial potential. Syzygium species have traditional practices to manage various illnesses, including mouth infections and oral thrush [8, 10]. Herein, the intended EOs have been extracted using three methods: hydrodistillation (HD), supercritical fluid (SF), and headspace (HS). Interestingly, the HD method has been previously implemented for the extraction of the EO from both species but from different geographical origins [38–41]. Conversely, this is the first report on the extraction of EOs from both species using state-of-the-art approaches such as supercritical fluid (SF), and head-space (HS). In general, the extraction conditions significantly affect the physical properties and the yield of an EO. As shown from our results, the SF method provided a higher oil yield than the conventional HD because the SF method utilizes supercritical CO_2_ as an extraction solvent. Supercritical CO_2_ is characterized by being a non-viscous solvent with low surface tension, thereby increasing the penetration rate, enhancing the extraction capacity, and amplifying the oil yield [42]. In addition, the solvation power of supercritical CO_2_ leads to the co-solubilization of some fatty constituents, which gives the SF extract a darker color and semi-solid consistency. Subsequently, the extracted EOs were analyzed qualitatively and quantitatively using hyphenated GC/MS, and the abundance of each constituent was estimated from the area of the peaks recorded by GC/FID. Our results showed that the identified volatile components were significantly variable among the two species concurrently with the applied extraction methods. This may be correlated to the differences in the condition of the plant material, the operated temperature, and pressure, in addition to the extraction time. In the SF method, dried leaves were extracted using supercritical CO_2_ (SCC) at 40 °C and 150 bar; in the HD method, fresh leaves were extracted using boiling water, while in the HS fresh leaves were heated until the volatilization of the aroma [21–23]. As depicted, the HD and HS utilized fresh leaves, while SF used dried ones which rationale the significant difference in the identified constituents of the SF EOs from the other two methods. Although HD and HS extracted the EO from a fresh plant sample, differences in the temperature and extraction time greatly affected the stability of the extracted constituents, nevertheless their detected percentage. A factor that may translate the significant differences we observed in the concentration of the identified major components from both methods. Also, the power of the extraction solvent, in each method, plays another crucial role. For instance, SCC behaves similarly to lipophilic solvents but with the advantage of adjustable selectivity. Hence, hydrocarbons are typically reported as majors in SF-EOs. On the other side, in the HD method, the diffusion of the boiling water inside the plant material enhanced the extraction of both oxygenated and non-oxygenated low molecular weight components. However, oxygenated components faced uncontrollable hydrolysis and decomposition due to the high temperature and long exposure time. Hence, HD-EOs were dominated by hydrocarbons and to a lesser extent stable, low molecular weight, oxygenated components [43, 44]. Ultimately, the HS technique is a relatively state-of-the-art extraction method that is designed to extract volatile constituents with a wide range of boiling points without developing artifacts [45]. Lastly, by skimming the available literature we deduced that the HD EOs showed variability in composition and yield from those reported in the literature. A remark that may be traced back to the environmental factors that the species were acclimatized to, such as seasonal conditions, and geographic sources, in addition to probable genetic discrepancies [3, 4].

Concerning the significance of the extracted EOs in oral health, the antimicrobial activity of the extracted EOs (HD and SF) was tested against four common oral pathogens, namely, *S. aureus*, *E. faecalis*, *E. coli*, and *C. albicans.* The microbes have been selected based on their *in-house* availability in addition to their pathogenic history in oral infection. For instance, *S. aureus* is considered a commensal as well as a human pathogen [46]. It is involved in several infective oral pathologies, including dental implant failure [47]. On the other side, *E. faecalis* plays an important role in human oral cavity infections such as endodontic infections, periodontitis, and peri-implantitis [15, 16, 48], while *E*. *coli* is considered a transitory microbiota in the oral cavity. It has been reported in acute dental abscesses cases, ranging from 0.7% to 15%. [19] (https://www.WHO.Int/news-room/fact-sheets/detail/e-coli). Ultimately, *C. albicans* is a commensal organism that lives in the oral cavity without causing any problems, but sometimes if the micro-environment changes it aggressively multiply and invade causing candidiasis in the mouth and throat (also called oral thrush) [20]. In the current study, three available assays were implemented to investigate the antimicrobial activity: the agar diffusion, the broth microdilution, and the biofilm formation. The agar diffusion assay is considered a rapid, simple, and prescreening qualitative method to assess the susceptibility of selected microbial strains and the degree of growth inhibition towards tested antimicrobial agents [49]. Herein, our results from the agar diffusion assay showed that the Gram-positive bacteria *S. aureus* and *E. faecalis* are the most susceptible organisms to the HD-EO of both *Syzygium species*, while *C. albicans* was sensitive only to *S. samarangense* HD oil. On the other side, the Gram-negative bacteria, *E. coli* showed resistant to most of the tested concentrations of both oils. One of the reasons that may explain this preference is the structural difference between Gram-positive and Gram-negative bacteria. Gram-positive bacteria possess a peptidoglycan layer that lies outside the bacterial outer membrane, whereas the outer membrane in Gram-negative bacteria, is composed of a double layer of phospholipids linked with lipopolysaccharides inner membrane [50]. Consequently, hydrophobic macromolecules in EOs become unable to penetrate the double membrane of the Gram-negative bacteria, so it develops instant resistance. Another factor that should be taken into consideration, is that even though the agar diffusion assay assumes that the antimicrobial agents diffuse freely in the solid nutrient medium [28], this assumption in many cases leads to significant deviations from the predicted results due to the variability among the volatile components during diffusion. To infer the activity level of our tested samples, we compare our results with the reported ZOI of HD-EOs from other S*yzygium* species. For instance, *S. aromaticum* essential oil (clove oil)*,* is traditionally renowned for treating toothache and a panel of oral infections [7, 8]. In agar diffusion assay, it displayed satisfactory antibacterial activity on *S. aureus* with measured ZOI of 12 mm at 100 mg/mL [51], while 12–20 mm with *E. faecalis* [52]. Also, the antibacterial activity of the HD EO from *S. cumini* leaves on *S. aureus* showed moderate ZOI of 12 mm at 10 μL/mL [53]. In conclusion, the HD EOs from our investigated species displayed acceptable preliminary antimicrobial effects compared to those reported in literature, though further investigation is needed.

Accordingly, the microdilution assay was implemented to provide higher accuracy and better coverage of the agar diffusion drawbacks [27, 28]. microdilution assay is the gold standard test for antimicrobial activity owed to its reproducibility, effective exposure to the tested antimicrobial samples, and the economy of reagents and tools [27, 28]. In this assay our investigated EOs displayed a promising ability to suppress the growth of all tested microbial strains in a dose-dependent manner. In addition, some microorganisms showed higher sensitivity to the tested EOs than the reference standard drug (Amoxicillin). For instance, the growth of *E. coli* and *C. albicans* was highly inhibited by the HD-EOs with MIC values being more potent than Amoxicillin. Concurrently, *E. faecalis* and *S. aureus* displayed almost double the MIC of *E. coli* and *C. albicans*, while still showing higher susceptibility to the administered HD EOs than the reference standard. Similarly, by comparing our results with those reported in the literature, the HD EO from *S. cumini* leaves displayed MIC values corresponding to 512 µg/mL and ≥ 1024 µg/mL on *E. coli* and *S. aureus,* respectively [54] compared to 3.5–15 μL/mL in our investigated oils*.* The HD EO of clove exhibited good antibacterial activity against *S. aureus* with a MIC of 0.625 mg/mL [55]. Hence, from the microdilution assay results, we could infer the promising broad-spectrum antimicrobial activity of the tested EOs which was undetectable by the agar diffusion assay. Secondly, the observed antimicrobial activity of the tested EOs is highly conserved, at least in part, to the chemical constituents in each oil. For instance, the HD EO of *S. malaccense* is abundant with oxygenated sesquiterpenes such as caryophyllene oxide. Reportedly, caryophyllene oxide possessed insecticidal and broad-spectrum antifungal activities [56, 57]. Thirdly, although some tested EOs showed similarities in their major composition, they possessed dissimilar MIC. The latter may be at least in part due to the variation in the percentage of every single component, the chance of synergism between the major components, or between the major and minor components [58, 59]. In this regard, *S. samarangense* HD EO that possessed potent MIC on *E. coli* (MIC = 3.7 μL/mL) constitutes considerable proportions of the terpenoids *p*-cymene (19.5%) and γ-terpinene (17.84%). Marchese et al. have mentioned that *p*-cymene alone with a concentration equal to 12 mg/mL completely inhibited the growth of *E. coli* [60]. In addition, Miladi and his research team have documented the synergistic action of *p*-cymene and γ-terpinene on the inhibition of drug-resistant bacteria [61]. So, synergism between EO components may increase the permeability of the plasma membrane or enhance their binding to transmembrane proteins, although the exact mechanism of action is often quite difficult to determine [62].

It is acknowledged that the phenotypic features of bacteria grown in biofilms are substantially distinctive from those grown in suspension. Because biofilms are the ordinary habitat for the great majority of oral bacteria, including those contributing to oral diseases. In this context, EOs were noticed in the literature as potent antibiofilm agents acting by diverse mechanisms. For instance, EOs exploit their hydrophobic nature in modifying the permeability of the cytoplasmic membrane with subsequent leakage of intracellular content or deactivation of the bacterial enzymes [63]. Also, EOs could block the quorum-sense system, inhibiting the transcription of flagellar genes, and modulating bacterial motility [64], while another study documented their ability in reducing bacterial adherence to inert surfaces [65]. Das and the research team have reported that EO enhanced the accumulation of reactive oxygen species, increasing oxidative stress, and causing cellular apoptosis [66]. Lastly, Fde et al. showed that EOs inhibit the ergosterol synthesis, a major constituent of the fungal plasma membrane [67]. Accordingly, it was deemed of interest to test the inhibition potential of the extracted EOs on biofilm formation. Interestingly, our results highlighted that the SF-EOs favorably inhibit the biofilm of Gram-positive bacteria rather than Gram-negative strains, while the HD-EOs are more potent to the *C. albicans* biofilm. Reportedly that oxygenated terpenes such as caryophyllene oxide and globulol, which are present in considerable amounts in the HD- and SF-EO of *S. malaccense* and *S. samarangense*, respectively, exert antimicrobial action by destroying the microbial cytoplasmic wall, improving its permeability, and allowing the passage of large protons and ions [68]. Moreover, previous reports have highlighted that squalene, the major component of *S. malaccense* SF-EO, possessed promising antimicrobial activity and antibiofilm formation against *S. aureus* and *E. coli* [69, 70]. Hence, we hypothesized that the wide variety of constituents in each EO is a positive factor that may limit the development of resistance which is a common issue for synthetic drugs [71]. In all, although many essential oils are generally considered safe, further investigations including toxicity studies and dosing in clinical settings are required .

## Conclusions

The EOs of *S.* *samarangense* and *S. malaccense* leaves were extracted using three different extraction methods: hydrodistillation (HD), supercritical fluid (SF), and headspace (HS), and their composition was compared qualitatively and quantitatively. The setup of each extraction method greatly impacts the profile of the extracted volatile components from the same species, while environmental and genetic factors may contribute to the variations of EOs between species. The SF method demonstrated significant advantages over the conventional HD method in terms of oil yield. The HD and HS volatile profiles revealed qualitative similarities while differed quantitatively. Caryophyllene oxide, *p*-cymene, and *γ*-terpinene were the abundant constituents in the HD EOs. They effectively inhibited the growth of Gram-negative bacteria and biofilm formation by *C. albicans*. The SF-EO of *S. malaccense* could be considered an economic source for squalene, while *S. samarangense* is for globulol (each constitutes > 50% of its oil), and they efficiently suppressed the biofilm formation of Gram-positive bacteria. *S.* *samarangense* and *S. malaccense* EOs are promising broad-spectrum antimicrobial hits. They are promoted for adjuvant formulation with other antimicrobial agents to improve the effectiveness of treating candidiasis, dental caries, and abscesses. However, further investigations are required especially to assess their toxicity and efficacy in clinical settings.

### Supplementary Information

**Additional file 1: Figure S1.** TIC of* Syzygium malaccense *leaves essential oil extracted by HD.** Figure S2.** TIC of *Syzygium malaccense *leaves volatile constituents  extracted by HS.** Figure S3.** TIC of* Syzygium malaccense *leaves essential oil extracted by SF.** Figure S4.** TIC of* Syzygium samarangense *leaves essential oil extracted by HD.** Figure S5.** TIC of* Syzygium samarangense *leaves volatile constituents extracted by HS.** Figure S6.** TIC of* Syzygium samarangense *leaves essential oil extracted by SF.

## Data Availability

All data generated or analyzed during this study are included in the manuscript and its supplementary information files.

## Abbreviations

EOs: Essential oils
GC/MS: Gas chromatography-Mass spectrometry
HS: Headspace
HD: Hydrodistillation
MIC: Minimum inhibitory concentration
SF: Supercritical fluid
ZOI: Zone of inhibition
