# Supplementary material for: Phytochemical profile and antimicrobial activity of essential oils from two Syzygium species against selected oral pathogens
Source: BMC Complement Med Ther. 2023 Dec 12;23:448. doi: 10.1186/s12906-023-04277-1 (PMC10714517; doi:10.1186/s12906-023-04277-1)
Supplement: Supplementary file 1 — Additional file 1: Figure S1. TIC of Syzygium malaccense leaves essential oil extracted by HD. Figure S2. TIC of Syzygium malaccense leaves volatile constituents extracted by HS. Figure S3. TIC of Syzygium malaccense leaves essential oil extracted by SF. Figure S4. TIC of Syzygium samarangense leaves essential oil extracted by HD. Figure S5. TIC of Syzygium samarangense leaves volatile constituents extracted by HS. Figure S6. TIC of Syzygium samarangense leaves essential oil extracted by SF. [file 12906_2023_4277_MOESM1_ESM.docx]

**Supplementary Figures**

**Figure S1: TIC of** ***Syzygium malaccense*** **leaves essential oil extracted by HD**

**Figure S2: TIC of *Syzygium malaccense* leaves essential oil extracted by HS**

**Figure S3: TIC of** ***Syzygium malaccense*** **leaves essential oil extracted by SF**

**Figure S4: TIC of** ***Syzygium samarangense*** **leaves essential oil extracted by HD**

**Figure S5: TIC of** ***Syzygium samarangense*** **leaves essential oil extracted by HS**

**Figure S6: TIC of** ***Syzygium samarangense*** **leaves essential oil extracted by SF**
